# Diagnostic role of shear wave elastography for differentiating benign and malignant breast masses

**DOI:** 10.4102/sajr.v24i1.1999

**Published:** 2020-12-21

**Authors:** Nichanametla Sravani, Ananthakrishnan Ramesh, Sathasivam Sureshkumar, Chellappa Vijayakumar, K.M. Abdulbasith, Gopal Balasubramanian, Pampa Ch Toi

**Affiliations:** 1 Department of Radiodiagnosis, Faculty of Health Science, Jawaharlal Institute of Postgraduate Medical Education and Research (JIPMER), Puducherry, India; 2 Department of Surgery, Faculty of Health Science, Jawaharlal Institute of Postgraduate Medical Education and Research (JIPMER), Puducherry, India; 3 Department of Pathology, Faculty of Health Science, Jawaharlal Institute of Postgraduate Medical Education and Research (JIPMER), Puducherry, India

**Keywords:** elastography, B-mode ultrasonography, BI-RADS, diagnostic value, non-invasive imaging, breast carcinoma

## Abstract

**Background:**

Use of B-mode ultrasound (US) may not obviate the need for diagnosis by histopathology, which is an invasive technique and remains the gold standard. These limitations are being overcome with the advent of shear wave elastography (SWE).

**Objectives:**

To assess the diagnostic role of SWE parameters and combined SWE and B-mode US in diagnosing malignant breast lesions.

**Method:**

This cross-sectional study included all patients with a breast mass on clinical examination. A B-mode US with a Breast Imaging Reporting and Data System (BI-RADS) assessment and SWE evaluation (distance ratio [DR], area ratio [AR] and shear wave velocity [SWV]) in the lesion and healthy breast tissue of all recruited patients was performed. Cut-offs for SWE parameters were derived by receiver operating characteristic (ROC) analysis. The diagnostic performance of the B-mode US, the SWE parameters and the combined imaging in diagnosing malignancy was assessed.

**Results:**

This study included a total of 175 breast masses. The median values of the SWE parameters were significantly higher (*p* < 0.001) in the malignant breast masses (DR, 1.29 vs. 1.03; AR, 1.69 vs. 1.06; and SWV, 9.1 metre per second [m/s] vs. 2.1 m/s). The ROC cut-off for malignancy was derived at 1.135 m/s, 1.18 m/s and 3.18 m/s, respectively, for DR, AR and SWV. The area under the ROC curve was highest for the DR (0.930), whilst this value was 0.914 and 0.901 for the SWV and AR, respectively. Amongst the respective sensitivities and specificities of the B-mode US (90.6% and 90%), SWE (97.6% and 61.1%), SWE (excluding AR) (96.5% and 77.8%) and combined imaging (100% and 72.2%), the highest sensitivity was noted for the combined method.

**Conclusion:**

All the SWE parameters were significantly higher in the malignant breast masses, compared to the benign lesions. On combining SWE and B-mode US, there was a significant increase in sensitivity but a decrease in specificity.

## Introduction

The foremost investigations used in the assessment of a breast mass are mammography and B-mode ultrasonography (US), depending on the patient’s age. These diagnostic methods have exhibited high sensitivity in diagnosing malignancy, but both have some limitations. The Breast Imaging Reporting and Data System (BI-RADS) classification score was formulated to standardise the mammography and US reporting system. However, that also generates a significant number of false positives, leading to an increase in biopsies with a cancer detection rate of 10% – 30%.^1,2^

Ultrasonography is the initial investigation of choice to differentiate between solid and cystic lesions of the breast. The limitation of US is its low specificity, owing to the solid nature of most benign lesions.^3^ As a result of these pitfalls, the use of B-mode US or mammography may not obviate the need for diagnosis by histopathology, which is an invasive technique and remains the gold standard.

Cancer tissues are harder than benign masses, even in the initial stages, because of the increased density of cells and blood vessels.^4^ Shear wave elastography (SWE) is a novel US technique that helps us to non-invasively assess the tissue stiffness and deformability, providing information on the elasticity of the mass. Combining US technology with the basic principles of SWE is indicating promising results in reducing the need for invasive diagnosis of breast masses and in limiting the negative biopsy rate.

Though a few studies have shown a high diagnostic performance for SWE parameters, other studies have shown that BI-RADS had significantly better accuracy than SWE.^5^ Additionally, there are not many studies on the role of SWE in breast tumours in the Indian context. This study was carried out to assess the diagnostic role of SWE parameters in the differentiation of benign and malignant breast masses compared to B-mode US.

## Methodology

This cross-sectional study was carried out in the Department of Radiology at a tertiary care hospital between 01 February 2015 and 30 November 2017. The research included all patients with a clinically palpable breast mass routinely referred to the Department of Radiodiagnosis for imaging. Male patients with breast masses were excluded to maintain homogeneity in the study population, as were patients who underwent biopsy or chemotherapy before imaging. Study participants with lesion sizes of <5 millimetres (mm) in the short axis or cystic lesions were excluded. Cases with inconclusive results on cytology or histology were not included.

B-mode US and SWE were performed using a Siemens Acuson S3000 US machine (Erlangen, Germany) by a group of three senior consultant radiologists who were not aware of previous investigation results. Blinding to B-mode US to avoid bias was not feasible, as the lesion’s localisation for SWE requires initial assessment by conventional B-mode US. The procedure was done with the patient lying down in a supine or supine oblique position using a linear (9L4) transducer that was equipped with acoustic radiation force impulse (ARFI) elastography functionality.

The findings recorded from the B-mode US included lesion size, shape, margin, orientation, posterior acoustic features, calcifications, axillary lymphadenopathy and internal vascularity. The BI-RADS categories were assigned according to the BI-RADS US lexicon, 5th edition, by the American College of Radiology in 2013.^6,7^ Breast Imaging Reporting and Data System categories 2 or 3 were considered as benign and categories 4 or 5 malignant.^6,8^

The elastography technique included qualitative, as well as quantitative techniques, including virtual touch imaging (VTI) and virtual touch quantification, and was carried out by the same radiologist after the B-mode US. The SWE was performed according to guidelines provided by Nakashima et al.^4^ and Lee et al.^9^

### Virtual touch imaging

After adequate visualisation of the mass by US, the probe was adjusted such that the B-mode image of the lesion was in the centre of the screen, and it was surrounded by adjacent normal tissue. Subsequently, elastography mode was activated to obtain a high-quality elasticity image. During the process, the probe was maintained perpendicular to the skin, and measures were taken to curtail manual compression. Essential care was also taken to adequately image the mass’s peripheral areas, as maximum stiffness in most of the malignant masses was noted in the peritumoral stroma. The shape of the lesion, distance ratio (DR) and area ratio (AR) were measured in the VTI elastogram. The shape of the lesion was described as oval, round or irregular. The DR was defined as the ratio of the mass diameters on the elastogram to the B-mode image. Similarly, the AR was the ratio of the areas of the mass on the elastogram to B-mode imaging.

### Virtual touch quantification

#### Shear wave velocity in the lesion

The shear wave velocity in the lesion (SWV [l]) was calculated as the mean value of SWV measurements taken from three independent acquisitions that fall under the 25 mm^2^ (5 mm × 5 mm) region of interest (ROI) box in the lesion. To obtain the SWV (l), a ROI was placed entirely in the breast lesion without including the adjacent parenchyma. Numerous measurements exceeded the upper limit of possible measures, indicated as ‘X.XX m/s’, which varies amongst different manufacturers (for the Siemens ACUSON S3000, X.XX denotes SWV ≥ 9.10 metres per second [m/s]). Therefore, in this study, we replaced X.XX m/s with a value of 9.10 m/s if it occurred in three consecutive measurements, after ruling out the patient’s respiration, movement and inappropriate placement of the ROI, as previously described in the literature by Bai et al.^10^ and Wojcinski et al.^11^

#### Shear wave velocity in normal breast tissue

Similar measurements were taken from normal breast tissue (SWV [n]) at the same depth but away from the lesion.

### Diagnostic utilisation of elastography parameters

The cut-off values for diagnosing malignancy were derived after plotting the receiver operating characteristic (ROC) curves for the DR, AR and SWV from our data. According to SWE, a mass was considered benign only when all the parameters were plotted within the range of benign masses. If any parameter was plotted above the cut-off, it was considered malignant. Thus, provisional diagnosis of the mass was made by SWE as benign or malignant.

The final diagnosis was established in all cases by tissue sampling (core needle biopsy; *n* = 100) or fine-needle aspiration cytology (FNAC; *n* = 75). The data regarding histopathology reports (HPR) were retrieved from the Hospital Information System.

Later, imaging (US, elastography or US combined with elastography) was compared with the pathological diagnosis (gold standard), and the necessary statistical tests were applied to assess the diagnostic role. The diagnostic performance of the elastography parameters was calculated according to the sensitivity, specificity, positive predictive value (PPV), negative predictive value (NPV) and area under the curve (AUC). The cut-off DR, AR and SWV values for maximal diagnostic accuracy were selected by considering the highest sum of sensitivity and specificity.

### Sample size calculation

The sample size was calculated assuming that the sensitivity and specificity of SWV for malignant breast tissues would be 95% with a 5% absolute precision and a 5% level of significance. The sample size (73 benign and 73 malignant) was estimated using the statistical formula for determining the sensitivity and specificity of a diagnostic tool.

### Statistical analysis

All the statistical tests were performed on SPSS software (version 19.0) from IBM. The Chi-square test or Fisher’s exact test was used to compare the categorical variables. Comparison of continuous variables between benign and malignant masses was carried out using the Mann–Whitney U-test or independent Student’s *t*-test, depending on the distribution of data. All statistical analyses were carried out at a 5% level of significance, where a *p*-value of < 0.05 was considered significant.

### Ethical consideration

The Institute Ethics Committee of Jawaharlal Institute of Postgraduate Medical Education and Research (JIPMER) (Human Studies; reg no. ECR/342/Inst/PY/2013) approved this study (approval no. JIP/IEC/SC/2014/8/647). The nature, methodology and risks of the study were explained to the patients, and informed consent was obtained. All the information collected was kept confidential, and patients were granted full freedom to withdraw during the study at any stage. All provisions of the Declaration of Helsinki were followed in this study.

## Results

In total, 175 breast masses from 163 patients were examined. Of these, 155 patients had single breast lesions; two breast lesions were noted in six patients, whilst the remaining two patients had four lesions. All the patients had HPR, either by FNAC or biopsy. The *t*-test indicated a statistically significant difference between the ages of women with benign and malignant breast masses (38.7 vs. 50.9, respectively; *p* ≤ 0.001). The most common symptom amongst the study participants was a breast lump. Most of the masses (45.1%) were located in the outer quadrant of the breast.

### Distribution of benign and malignant lesions in the study population

About 51% of breast masses (*n* = 90) were benign. Fibroadenoma (58%) and infiltrating ductal carcinoma (IDC) (86%) were the most common benign and malignant lesions, respectively. Eighty-five lesions were malignant on HPR, constituting about 49% of the study population. Most of the benign masses (*n* = 75), representing approximately 83.3% of the sample, were diagnosed by FNAC. Only 16.7% of the benign masses (*n* = 15) and all malignant lesions had undergone core needle biopsy for the final diagnosis (*n* = 85).

### B-mode ultrasonography parameters between benign and malignant groups

**FIGURE 1:**
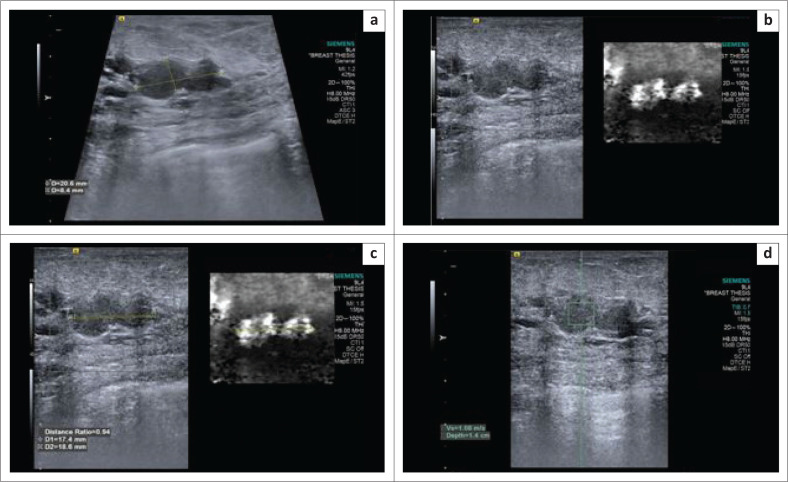
(a) B-mode ultrasonography image demonstrating a hypoechoic, lobulated lesion oriented parallel to the skin; (b) virtual touch imaging elastogram showing the lesion to be bright in comparison to the adjacent breast parenchyma, suggesting a soft lesion; (c) distance ratio of < 1 (0.94); (d) shear wave velocity in the lesion – 1.66 m/s. The final histopathology report was a fibroadenoma.

**FIGURE 2:**
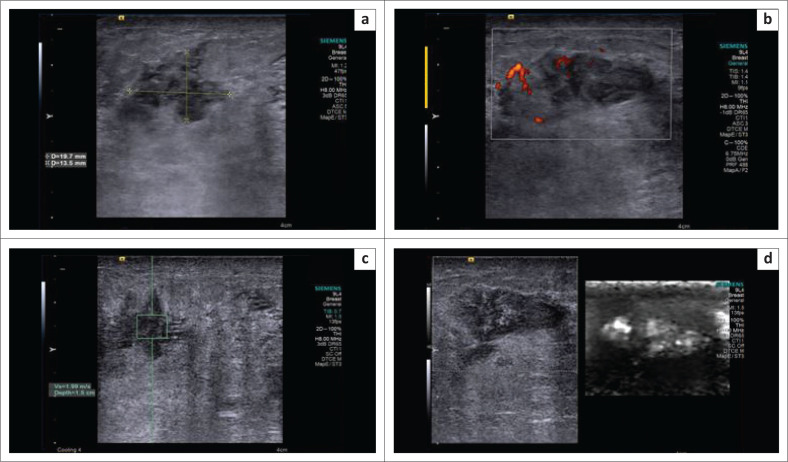
(a) B-mode ultrasonography image indicating an irregular, isoechoic spiculated lesion suggesting a Breast Imaging Reporting and Data System 4 lesion; (b) on power Doppler, the lesion demonstrates internal vascularity; (c) shear wave velocity in the lesion was 1.99 m/s; (d) the lesion appeared bright on virtual touch imaging elastogram, suggestive of a soft lesion. The final histopathology report was granulomatous mastitis.

The majority of malignant masses had spiculation (97.5%), ill-defined margins (95.2%), taller than wider orientation (76.2%), irregular shape (72.3%) and calcifications (72.2%). Similarly, masses with a well-defined margin (91.1%), oval shape (84.1%) and wider than taller orientation (62.5%) were benign. Parameters like the size, shape, orientation, margins, posterior acoustic features, internal vascularity of the lesions and axillary lymphadenopathy patterns were significantly different amongst the benign and malignant breast masses (*p* ≤ 0.001). Axillary lymphadenopathy with loss of the fatty hilum was associated with a higher proportion of malignancy. See Table 1 and Figures 1 and 2.

**TABLE 1:** Distribution of B-mode ultrasonography parameters between benign and malignant groups.

Characteristic(s) of B-mode US	Variable	*n*	Final diagnosis				*p*†	Chi-square value
			Malignant (*n* = 85; 100%)		Benign (*n* = 90; 100%)			
			Total	%	Total	%		
Mean size (cm)	**-**	-	2.34	-	2.77	-	0.03‡	-
Shape	-	-	-	-	-	-	< 0.001	53.765
	Irregular	101	73	72.3	28	27.7	-	-
	Oval	69	11	15.9	58	84.1	-	-
	Circular	5	1	20.0	4	80.0	-	-
Orientation of mass	-	-	-	-	-	-	< 0.001	19.589
	Wider than tall	120	45	37.5	75	62.5	-	-
	Taller than wide	42	32	76.2	10	23.8	-	-
	Not applicable	13	8	61.5	5	38.5	-	-
Margins of lesion	-	-	-	-	-	-	< 0.001	55.923
	Well-defined	114	32	28.8	82	71.9	-	-
	Partly defined	40	33	82.5	7	17.5	-	-
	Ill-defined	21	20	95.2	1	4.8	-	-
Margin characteristics	-	-	-	-	-	-	< 0.001	64.734
	Lobulations	89	41	46.1	48	53.9	-	-
	Spiculations	40	39	97.5	1	2.5	-	-
	Smooth	46	5	10.9	41	89.1	-	-
Posterior acoustic shadowing	-	-	-	-	-	-	< 0.001	42.946
	Present	36	35	97.2	1	2.8	-	-
	Absent	139	50	36.0	89	64.0	0.03	-
Calcifications	-	-	-	-	-	-	-	4.493
	Present	18	13	72.2	5	27.8	-	-
	Absent	157	72	45.9	85	54.1	-	-
Axillary lymphadenopathy	-	-	-	-	-	-	< 0.001	-
	With fatty hilum	80	21	26.2	59	73.8	-	-
	Lost fatty hilum	87	58	66.6	29	33.3	-	-
	Eccentric hilum	8	6	75.0	2	25.0	-	-
Internal vascularity	-	-	-	-	-	-	< 0.001	81.739
	Present	95	66	69.5	29	30.5	-	-
	Absent	80	19	23.7	61	76.3	-	-

US, ultrasonography.

† , Used chi-square test;

‡ , Independent Student’s *t*-test.

### Elastography parameters between benign and malignant masses

**FIGURE 3:**
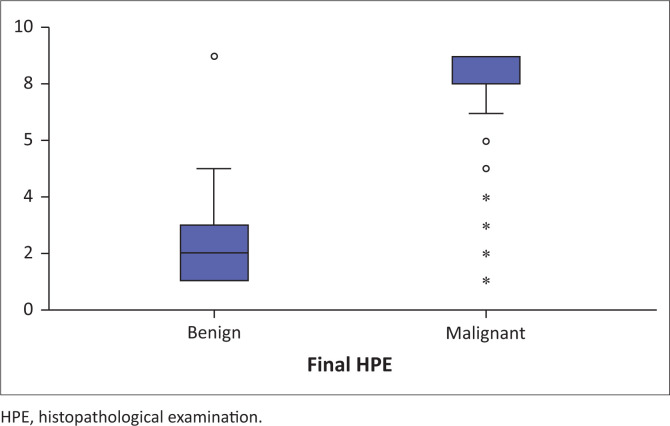
Box plot showing shear wave velocity in benign and malignant breast masses.

All the SWE parameters were higher in the malignant breast masses than in the benign lesions. The median DR, AR and SWV (l) in the malignant and benign masses were 1.29 m/s versus 1.03 m/s, 1.69 m/s versus 1.06 m/s and 9.1 m/s versus 2.1 m/s, respectively, with a statistically significant difference (*p* < 0.001) (Figure 3). The median SWV (n) in the normal breast tissue of patients with benign and malignant masses was statistically significant (1.26 vs. 1.46; *p* = 0.03). However, the difference in numerical values is minimal in Table 2. There were a few technical difficulties during the study, which included the inability to measure AR accurately for benign masses and obtaining X.XX m/s as the SWV for the majority of malignant masses.

**TABLE 2:** Distribution of elastography parameters between benign and malignant lesions.

Elastography parameter	Median				p*
	Malignant		Benign		
	Median	IQR	Median	IQR	
Distance ratio	1.29	1.20–1.44	1.03	1.00–1.09	< 0.001
Area ratio	1.69	1.39–1.93	1.06	1.01–1.18	< 0.001
SWV (l), m/s	9.10	8.35–9.1	2.10	1.47–2.78	< 0.001
SWV (n), m/s	1.46	1.11–2.04	1.26	0.91–1.85	0.03

IQR, interquartile range; SWV (l), shear wave velocity in the lesion; SWV (n), shear wave velocity in normal breast tissue.

* , Used Mann–Whitney U-test.

### Comparison of shear wave velocity in various subgroups of benign and malignant breast masses

Of all the benign lesions, the mean SWV was lowest in the case of lipoma and highest for ductal hyperplasia. The mean SWVs for fibroadenoma, phyllodes tumour and benign proliferative breast disease were 2.29 m/s, 2.15 m/s and 1.96 m/s, respectively. Papillary carcinoma showed the lowest mean, as well as a median SWV of 1.31 m/s. Adenoid cystic carcinoma and malignant phyllodes tumour also showed lower SWVs of 3.19 m/s and 2.54 m/s in comparison to IDC. In IDC, Grade III showed the lowest SWV and showed high variability in SWV.

### Cut-off values and diagnostic performance of individual elastography parameters

**FIGURE 4:**
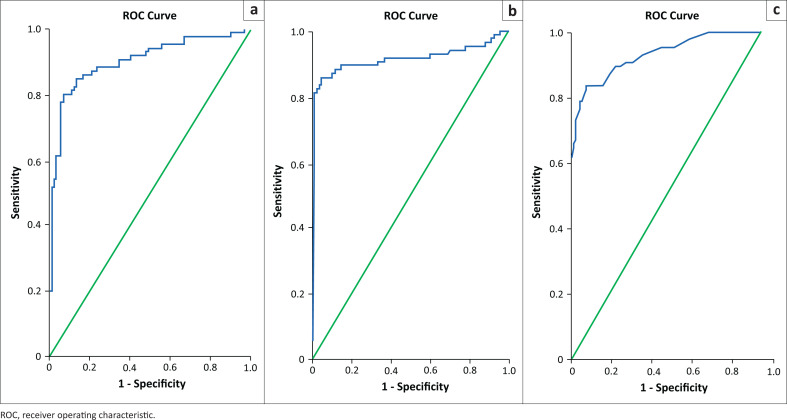
Receiver operating characteristic curve for (a) shear wave velocity, (b) distance ratio and (c) area ratio.

The DR had the highest PPV amongst the elastography parameters for defined cut-off, approximately 91%. The highest sensitivity and NPV were obtained for SWV, whilst the DR showed the highest specificity and PPV. Evaluating SWV with a ROC analysis for identifying a malignant lesion yielded a sensitivity of 89.4% and specificity of 85.6% when a cut-off SWV of 3.18 m/s was defined. However, if a cut-off SWV of 3.43 m/s was considered, the sensitivity and specificity were 87.1% and 90%, respectively (Table 3 and Figure 4).

**TABLE 3:** Cut-off values and diagnostic performance of individual elastography parameters.

Parameter	AUC	Cut-off (m/s)	Sensitivity (%)	Specificity (%)
DR	0.930	≥ 1.135	83.5	92.2
AR	0.901	≥ 1.18	88.2	76.7
SWV (l)	0.914	≥ 3.18	89.4	85.6
SWV (n)	0.914	≥ 3.433	87.1	90.0

AUC, area under the curve; AR, area ratio; DR, distance ratio; SWV (l), shear wave velocity in the lesion; SWV (n), shear wave velocity in normal breast tissue.

### Diagnostic performance of various imaging modalities and elastography parameters

Considering all three elastography parameters, DR, AR and SWV, with respective cut-off values for diagnosing malignancy, the sensitivity and specificity were 97.6% and 61.6%, respectively. Similar diagnostic accuracy but better specificity was achieved when AR was excluded and the study considered only DR and SWV (l) for diagnosis (96.5% and 77.8%, respectively). The highest sensitivity and NPV of 100% was obtained for combined imaging utilising B-mode US and SWE. The highest specificity was for B-mode US and SWV with a cut-off value of 3.43 m/s (90%). The SWV (l) showed the highest PPV of 90.7% (Tables 4 and 5).

**TABLE 4:** Comparison of ultrasonography and elastography parameters in the diagnosis of benign and malignant breast lesions.

Diagnostic performance	Final radiological diagnosis	Total	%	Final pathological diagnosis			
				Malignant (*n* = 85; 100%)		Benign (*n* = 90; 100%)	
				Total	%	Total	%
US diagnosis	Malignant	86	50.9	77	90.6	9	10.0
	Benign	89	49.1	8	9.4	81	90.0
Elastography (175) (DR, AR and SWV)	Malignant	118	67.4	83	97.6	35	38.9
	Benign	57	32.6	2	2.4	55	61.1
Elastography (175) (DR and SWV)	Malignant	102	58.3	82	96.5	20	22.2
	Benign	73	41.7	3	3.5	70	77.8
B-mode US and Elastography (175)	Malignant	110	62.9	85	100.0	25	27.8
	Benign	65	37.1	0	0.0	65	72.2

AR, area ratio; DR, distance ratio; SWV, shear wave velocity; US, ultrasonography.

**TABLE 5:** Diagnostic performance of various imaging modalities.

Imaging modality	Sensitivity (%)	Specificity (%)	PPV (%)	NPV (%)
B-mode US-based BI-RADS	90.6	90.0	89.5	91.0
Elastography (DR, AR and SWV)	97.6	61.1	70.3	96.5
Elastography (DR and SWV)	96.5	77.8	80.4	95.9
Elastography (SWV – 3.18 m/s)	89.4	85.6	85.4	89.5
Elastography (SWV – 3.43 m/s)	87.1	90.0	90.7	86.5
B-mode US and elastography	100	72.2	77.3	100.0

AR, area ratio; BI-RADS, Breast Imaging Reporting and Data System; DR, distance ratio; NPV, negative predictive value; PPV, positive predictive value; SWV, shear wave velocity; US, ultrasonography.

## Discussion

In the present study, all the SWE parameters were significantly higher in malignant breast masses as compared to the benign lesions. A significant increase in sensitivity was found when combining SWE features with B-mode US findings; however, the specificity decreased considerably. Lesions with an irregular shape, spiculations, taller than wider orientation, posterior acoustic shadowing, calcifications and internal vascularity favoured the malignant nature of lesions. Oval-shaped lesions with smooth margins, wider than taller lesions and the absence of internal vascularity favoured benignity. These findings are comparable to the results obtained in previous studies by Stavros et al.^12^ and Mainiero et al.^13^ The presence of axillary lymphadenopathy with absent or a compressed fatty hilum was very specific for malignant masses.

**FIGURE 5:**
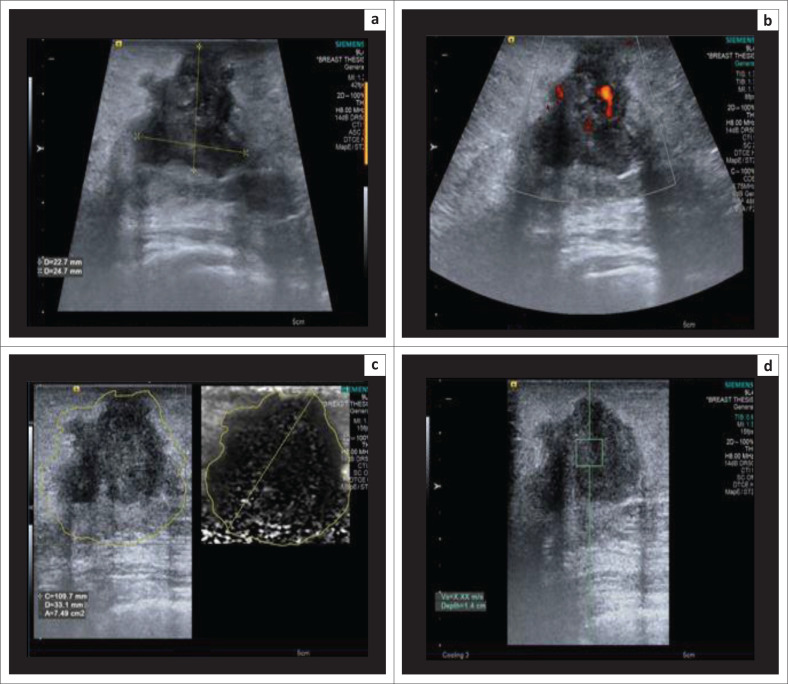
(a, b) Greyscale image showing a classical Breast Imaging Reporting and Data System 5 lesion – an irregular, spiculated, taller than wide lesion with a colour Doppler image showing internal vascularity; (c) elastogram showing a hard lesion (grey to black) with a larger area of the lesion on elastogram as compared to B-mode ultrasonography; (d) shear wave velocity in the lesion – X.XX m/s. The final histopathology report was infiltrating ductal carcinoma.

Malignant masses appeared larger on elastograms because of infiltration into the adjacent parenchyma, and hence higher DR and AR values were obtained. They were hard because of the high density of cells and the desmoplastic reaction, appearing dark on elastograms. Furthermore, shear waves are produced when the US-generated push pulse hits a hard mass and travels faster. Accordingly, malignant masses showed higher SWVs (Figure 5). Benign masses were soft, bright and either of equal size or decreased size on elastograms compared to the B-mode US (Figures 1 and 2). Difficulty in estimating DR and AR occurred in big malignant masses as the elastographic overlay could not entirely cover the large masses.

Approximately 74.1% of the malignant masses (*n* = 63) showed X.XX m/s as the SWV in the lesion on three consecutive measurements, and hence their real stiffness could not be measured. Nakashima et al.^4^ have stated that the absolute value of SWV in such cases could be obtained by placing an ROI in the mass’s periphery and including adjacent breast tissue, but this was not undertaken in this study.

The ROC analysis for elastography parameters showed a significantly high AUC. These cut-off values were comparable to those obtained in previous studies by Berg et al.^8^ and Bai et al.^11^ The SWV cut-offs were defined at 3.18 m/s and 3.43 m/s. Previous studies done over the past decade by Meng et al.,^14^ Wojcinski et al.,^10^ Kim et al.,^15^ Tang et al.^16^ and Bai et al.^11^ showed varying cut-off velocities ranging between 3.05 m/s and 9.1 m/s. The differences could be the result of diversity in the distribution of malignant and benign masses in their study population.

**FIGURE 6:**
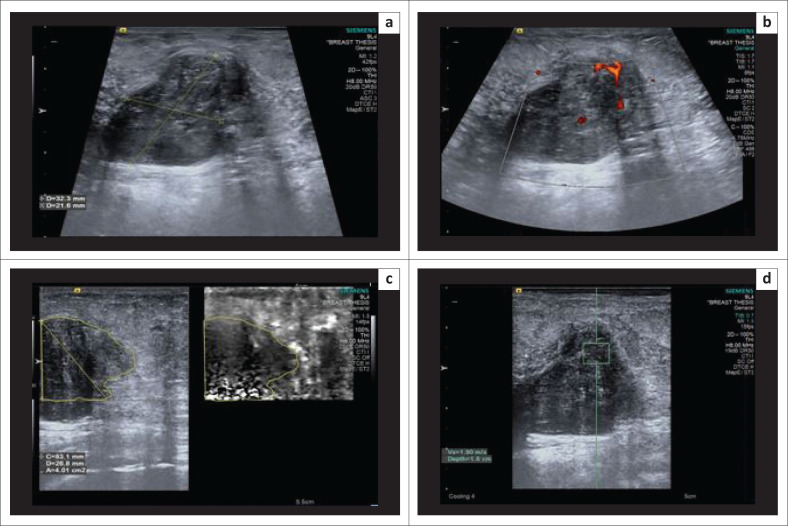
(a, b) B-mode ultrasonography demonstrating an oval, lobulated, taller than wide, heterogeneously hypoechoic lesion with internal vascularity on power Doppler, indicating a Breast Imaging Reporting and Data System 4 lesion; (c) elastogram indicating a relatively soft lesion with no obvious extension of the lesion into the adjacent parenchyma, with an area ratio < 1; (d) shear wave velocity in the lesion was 1.90 m/s. The final histopathology report was benign phyllodes tumour.

Benign phyllodes tumour (*n* = 8) and infective lesions (*n* = 3) were wrongly categorised as malignant (BI-RADS 4) on the B-mode US because of their irregular shape, heterogeneous echotexture and significant vascularity. However, elastography correctly categorised them as benign, as they demonstrated lower DR, AR and SWV in the benign range (Figure 6). In these cases, a biopsy could be avoided with the addition of elastography features. One case of malignant phyllodes tumour existed in the present study population, which showed lower DR, AR and SWV. Hence, elastography’s ability to differentiate benign phyllodes tumour from its malignant counterpart has to be further tested, with the inclusion of an adequate number of benign and malignant forms.^17^

**FIGURE 7:**
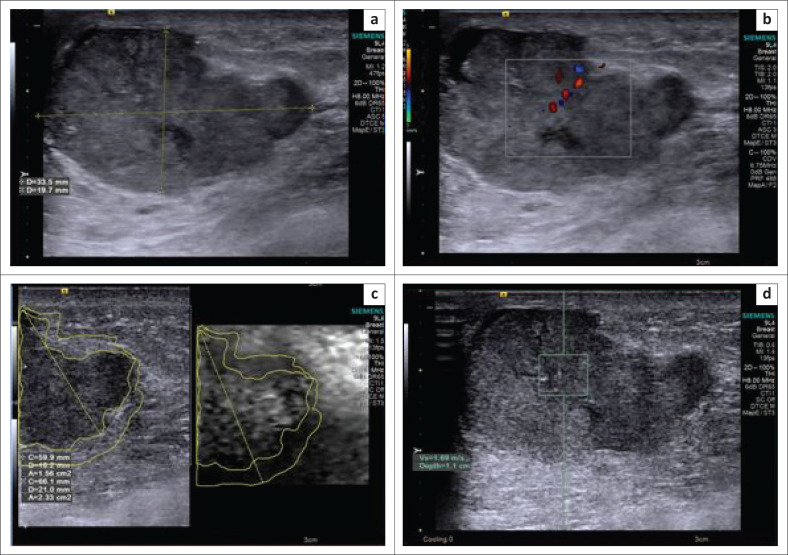
(a, b) B-mode ultrasonography image of an irregular, hypo- to isoechoic and predominantly solid lesion with few anechoic areas and internal vascularity on colour Doppler, suggesting a Breast Imaging Reporting and Data System 4 lesion; (c) elastogram showing softer areas in the centre of the lesion with a relatively firm periphery; (d) shear wave velocity in the lesion – 1.69 m/s. The final histopathological diagnosis was papillary carcinoma.

A few malignant lesions indicated a SWV less than the derived cut-off SWV for diagnosing malignancy (*n* = 9). Retrospective analysis of these cases showed a significant proportion of soft areas on ARFI imaging (*n* = 5). This could be attributed to the necrotic nature of the tumour. Furthermore, in papillary carcinoma, SWV was lower because of the tumour characteristics (Figure 7).^18^

There were technical difficulties in obtaining the AR for some benign masses because of poor delineation of the mass on the elastogram. This occurred because the elasticity of the lesion resembled that of the adjacent breast parenchyma (Pattern 2, according to Tozaki et al.).^19^ This has led to a spuriously high AR value for a few benign lesions, leading to the low specificity of 76.7%. This is in contrary to a previous study by Bai et al.^11^, which showed better specificity for the AR over the SWV. Whilst the current study demonstrated a poor diagnostic performance for the AR, Bai et al.^11^ achieved a high specificity for the AR by excluding the AR for masses that followed Pattern 2 on ARFI imaging. They described the diagnostic performance for AR as the best amongst all the elastography parameters.

With the combination of B-mode US and elastography parameters, similar to a previous study by Zhou et al.^20^, this study has obtained 100% sensitivity and NPV in diagnosing breast malignancy. With the combined modality, this study has shown a significant increase in sensitivity, from 90.6% to 100%. However, the specificity fell significantly, from 90% by B-mode US to 72.2% with combined imaging. This was contradictory to the results obtained in previous studies by Kim et al.^8^ and Berg et al.^15^, who showed an increase in specificity by adding SWE features to B-mode US-based BI-RADS analysis. This is the result of the low specificity of AR amongst elastography parameters in this study. Using a combination of imaging modalities for diagnosis, we can select 100% of malignancies accurately for biopsy.

### Limitations

Initially obtaining greyscale images from the B-mode US was inevitably required for conducting elastography. Hence, bias could have occurred in the evaluation of the elastography features. The sample size for many histological subgroups in benign and malignant breast masses was small. Further analysis is needed to evaluate elastography’s diagnostic role in detecting malignant phyllodes tumours and malignant foci in ductal papillomatosis.

The subcategorisation of BI-RADS 4 was not carried out in the present study; elastography features for stratification of BI-RADS may add more information on the usefulness of breast elastography. Both the conventional and ARFI assessments were performed with a Siemens Acuson S3000 US machine using a 9L4 linear transducer (4 megahertz [MHz] – 9 MHz). The 6L18 (18 MHz) probe used for a sonogram at our institute did not have ARFI elastography, and therefore, to carry out the sonogram and elastography together, the 9 MHz linear transducer was used. A linear probe with 14 MHz, or above, may yield better imaging for breast masses.

## Conclusion

Elastography may be considered as part of the routine workup for breast masses to improve the sensitivity for diagnosing malignant lesions and avoiding false-negative reports. It can be used as an adjunct tool in characterising a breast mass, especially in suspicious appearances on the B-mode US, to downgrade or upgrade lesions accordingly. It is also useful in determining the need for performing an invasive histopathological examination and avoiding unwarranted biopsy in some benign breast lesions.
